# Gemcitabine Induces Poly (ADP-Ribose) Polymerase-1 (PARP-1) Degradation through Autophagy in Pancreatic Cancer

**DOI:** 10.1371/journal.pone.0109076

**Published:** 2014-10-01

**Authors:** Yufeng Wang, Yasuhiro Kuramitsu, Kazuhiro Tokuda, Byron Baron, Takao Kitagawa, Junko Akada, Shin-ichiro Maehara, Yoshihiko Maehara, Kazuyuki Nakamura

**Affiliations:** 1 Department of Biochemistry and Functional Proteomics, Yamaguchi University Graduate School of Medicine, Ube, Yamaguchi, Japan; 2 Department of Surgery and Science, Graduate School of Medical Science, Kyusyu University, Fukuokashi, Fukuoka, Japan; 3 Centre of Clinical Laboratories in Tokuyama Medical Association Hospital, Shunan, Japan; Johns Hopkins University, United States of America

## Abstract

Poly (ADP-ribose) polymerase-1 (PARP-1) and autophagy play increasingly important roles in DNA damage repair and cell death. Gemcitabine (GEM) remains the first-line chemotherapeutic drug for pancreatic cancer (PC). However, little is known about the relationship between PARP-1 expression and autophagy in response to GEM. Here we demonstrate that GEM induces DNA-damage response and degradation of mono-ADP ribosylated PARP-1 through the autophagy pathway in PC cells, which is rescued by inhibiting autophagy. Hypoxia and serum starvation inhibit autophagic activity due to abrogated GEM-induced mono-ADP-ribosylated PARP-1 degradation. Activation of extracellular regulated protein kinases (ERK) induced by serum starvation shows differences in intracellular localization as well as modulation of autophagy and PARP-1 degradation in GEM-sensitive KLM1 and -resistant KLM1-R cells. Our study has revealed a novel role of autophagy in PARP-1 degradation in response to GEM, and the different impacts of MEK/ERK signaling pathway on autophagy between GEM-sensitive and -resistant PC cells.

## Introduction

Gemcitabine (GEM) is currently the standard treatment for advanced and metastatic pancreatic cancer (PC) in both adjuvant and palliative settings, but resistance to GEM has been a big problem as its response rate has been reduced to <20% [1]–[4]. GEM can inhibit DNA synthesis by targeting ribonucleotide reductase, leading to its inclusion into cellular DNA, causing DNA replication errors [5], [6]. A previous study has reported that GEM-induced DNA replication stress, stalled replication forks and triggered checkpoint signaling pathways [7]. Inhibition of checkpoint kinase 1 (Chk1) with chemical inhibitors induced sensitization of PC cells in response to GEM [8], [9]. Moreover mismatch repair-deficient HCT116 cells are more sensitive *in-vitro* to GEM-mediated radiosensitization [8]. Although the evidence has shown the relationship between DNA repair and sensitization of cells to GEM, the mechanisms responsible for the repair of GEM-induced DNA damage are not clearly understood.

Autophagy is a cellular pathway involved in the routine turnover of proteins or intracellular organelles with close connections to human disease and physiology [10]. Autophagic dysfunction is associated with cancer, neurodegeneration, microbial infection and as well as resistance of cancer cells to anticancer therapy [11], [12]. GEM induced autophagy in Panc-1 and MiaPaCa-2 cells, and inhibition of autophagy by 3-methyladenine (3-ME) or vacuole membrane protein 1 knockdown decreased apoptosis in gemcitabine-treated cells [13]. Therefore this evidence indicates that autophagy may play an essential role in apoptosis of PC cells in response to GEM.

Poly (ADP-ribose) polymerase-1 (PARP-1) plays critical roles in many molecular and cellular processes, including DNA damage repair, genome stability, transcription and apoptosis [14]. PARP1 is involved in the repair of both single-stranded DNA (ssDNA) and double-strand DNA (dsDNA) breaks by binding with DNA ends and/or interacting with DNA repair proteins, example (Ataxia Telangiectasia Mutated) ATM and Ku subunits [15]–[18]. Inhibition of PARP-1 enhances the cytotoxicity of DNA-damaging agents and radiation *in-vitro* [19]. PARP-1 inhibitors have been reported as potential chemotherapeutic drugs for BRCA1/BRCA2-deficient breast cancer and lung cancer [20]–[21]. Thus it is necessary to assess the changes of PARP-1 responsible for GEM-induced DNA damage in PC.

In the present study, we demonstrate that microtubule-associated protein 1A/1B-light chain 3 (LC3), a key factor of autophagosome formation, is down-regulated in KLM1-R compared to KLM1 cells. GEM induced a DNA damage response and autophagy in KLM1 and KLM1-R cells and down-regulated PARP-1 expression. Inhibition of autophagy blocked GEM-induced degradation of mono-ADP ribosylated PARP-1. The MEK/ERK signaling pathway showed a different effect on autophagy and GEM-induced PARP-1 degradation between KLM1 and KLM1-R cells. Thus we highlight new insight regarding the autophagy pathway in regulating PARP-1 degradation in PC cells.

## Material and Methods

### Materials

U0126 (9903S) and wortmannin (9951S) were purchased from Cell Signaling Technology. The antibodies specific for p-ERK (sc-7383), ERK (sc-94200), p21 (sc-65595), Hsp27 (sc-13132), PARP-1 (sc-8007 for western blot and sc-1562 for confirmation and immunofluorescence), CtIP (sc-3970) and actin (sc-1616) were purchased from Santa Cruz Biotechnology. The antibodies specific for LC3A/B (4108S), SIRT6 (12486), caspase-3 (9665) and PI3KCIII (4263S) were purchased from Cell Signaling Technology. The antibodies specific for Bcl2 (B3170), Ulk1 (SAB4200106) and Beclin1 (B6061) were purchased from Sigma. The antibodies specific for AMPKα1 (07–350) was purchased from Millipore.

### Cell culture

All the cell lines used in this study were previously published cell lines that were provided to us as a gift. Human pancreatic cell line KLM1 (ID: TKG0490) was cloned and established by Dr. Kobari M from the Institute of Department, Aging and Cancer, Tohoku University (Sendai, Japan) in 1996 [39]. GEM-sensitive KLM1 and -resistant KLM1-R human pancreatic cancer cell lines were generously provided as a gift by the Department of Surgery and Science at Kyushu University Graduate School of Medical Science. KLM1-R has been established exposing KLM1 cells to GEM in previous studies [40]–[41]. These cells were cultured in Roswell Park Memorial Institute 1640 medium (RPMI 1640; GIBCO, 05918), supplemented with 10% heat-inactivated fetal bovine serum (FBS; GIBCO, 26140–079), and 2 mM L-glutamine and incubated at 37°C in a humidified incubator containing 5% CO_2_.

### Transient transfection

KLM1 cells were seeded and incubate at 37°C in a CO_2_ incubator until the cells are 70% confluent. The cells were transfected with validated human LC3B siRNA (sc-43390, Santa Cruz Biotechnology) or control siRNA (sc-37007, Santa Cruz Biotechnology) by following a siRNA Transfection Protocol (Santa Cruz Biotechnology).

### Western blotting

The cells were lysed with lysis buffer ((1% NP-40, 1 mM sodium vanadate, 1 mM PMSF, 50 mM Tris, 10 mM NaF, 10 mM EDTA, 165 mM NaCl, 10 µg/mL leupeptin, and 10 µg/mL aprotinin) on ice for 1 h. Cell lysates were then centrifuged at 15,000×g for 20 min at 4°C. The supernatant was collected and the protein concentration was determined by Lowry assay. Equal amounts of protein (20 µg) were resolved by 5–20% SDS-polyacrylamide gel and then transferred onto PVDF membrane (Immobilon-P; Millipore, Bedford, MA). The membrane was incubated with the appropriate primary antibody at 4°C overnight. Then, the membrane was washed and incubated with a horseradish peroxidase (HRP)-conjugated secondary antibody for 1 h at room temperature. The immunoblots were visualized with a chemiluminescence reagent (Immunostar, Wako). All of experiments were repeated for three times.

### Immunofluorescence

Cells were cultured on 15 mm round coverslips in 12 well plates at a density of 1×10^5^ cells per well. Cells on the coverslips were fixed using fresh 3.7% paraformaldehyde in phosphate buffered saline (PBS) for 30 min when they reached 70–80% confluency. Samples were then washed with PBS, followed by permeabilization with 0.1% Triton X-100 for 15 min. After washing with PBS they were incubated in blocking solution (1% goat serum or 1% donkey serum in PBS with 0.1% Tween 20) for 1 h at room temperature. Cells were treated with a primary antibody in blocking solution overnight at 4°C. After incubation with primary antibody, cells were rinsed with PBS with 0.1% Tween 20 (PBS-T) and incubated with a secondary antibody for 1 h at room temperature. After washing with PBS-T, their nuclei were counter-stained with 1.43 µM DAPI (4,6′-diamidino-2-phenylindole) for 5 minutes. Coverslips were washed with PBS-T, then mounted face-down onto microscope slides with Fluoromount (Diagnostic BioSystems, Pleasanton, CA, USA). Confocal images were obtained using Nikon Plan Apo 60X/1.40 objective, BZ-9000 series (BIOREVO) and BZ-II Viewer software (Keyence, Osaka, Japan) by an operator who was unaware of the experimental condition. All parameters were kept constant within each experiment. Digital images were analyzed and the average intensity was measured using Image J. software.

### Apoptosis assay

An appropriate number of cells was plated and treated for 24 h. Cells were stained by using Apo-BrdU *In Situ* DNA fragmentation Assay kit (80101, Biovision, Inc.) (data not shown) or Caspases 3/7 assay kit (12D51, ImmunoChemistry Technologies, LLC.). These experiments were performed strictly following the instructions of the relative protocols.

## Results

### Gemcitabine (GEM) induces autophagy in PC cells

**Figure 1:**
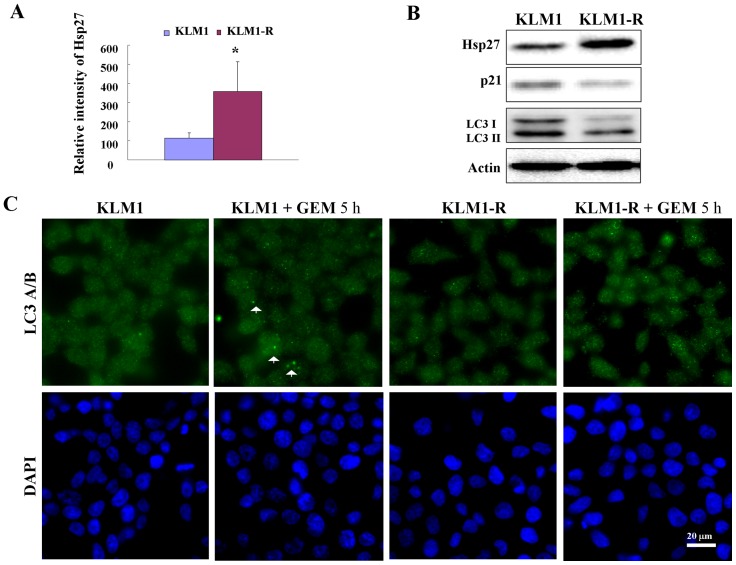
GEM induces autophagy in PC cells. (A) The expression of Hsp27 was tested by western blot and the relative intensity was measured by student-*t* test (n = 3) (B) KLM1 and KLM1-R cells were lysed and resolved by SDS-PAGE and probed with specific antibodies. Actin was used to normalize the loading levels of protein. (C) The indicated cells were treated with 100 μg/mL of GEM for 5 h and the expression of LC3A/B and formation of LC3-positive autophagosomes were examined by confocal microscopy. LC3A/B: green and DAPI: blue. Arrows indicate the autophagosome. Scale bar, 20 μm.

Two PC cancer cell lines GEM-sensitiive KLM1 and -resistant KLM1-R, were used in this study. These cell lines are defined by their expression of heat shock protein 27 (Hsp27) (Fig. 1 A and B), which has been reported as a potential marker for PC-resistant to GEM [22]–[24]. Moreover the expression of p21 was shown to be reduced in KLM1-R compared to KLM1 cells (Fig. 1 B), indicating the different phenotypes of cell cycle between them. We then investigated autophagic activity in KLM1 and KLM1-R cells, which was determined by the expression of LC3 [25]. We demonstrated that both LC3-I and II were down-regulated in KLM1-R compared to KLM1 cells (Fig. 1 B). Moreover, down-regulation of AMP-activated protein kinase A1 (AMPKα1) and unc-51-like kinase 1 (Ulk1) were shown, unlike phosphatidylinositol 3- kinase (PI3K CIII) or Coiled-coil myosin-like BCL2-interacting protein (Beclin-1), in KLM1-R compared to KLM1 cells (Fig. S1 A and B), indicating that the reduction of autophagic activity in GEM-resistant KLM1-R cells may be related to the down-regulation of AMPKα1 and/or Ulk1 expression. To determine the effect of autophagy induced by GEM, cells were treated with GEM for 5 hours (h) and then observed by immunofluorescent microscopy using anti-LC3 antibody staining. In this experimental setting, we demonstrated that the LC3 II spots were increased after the cells were exposed to GEM (Fig. 1 C). These data suggested that GEM induces autophagy in PC cells.

### GEM specifically down-regulates mono-ADP ribosylated PARP-1 in a caspase-independent manner

**Figure 2:**
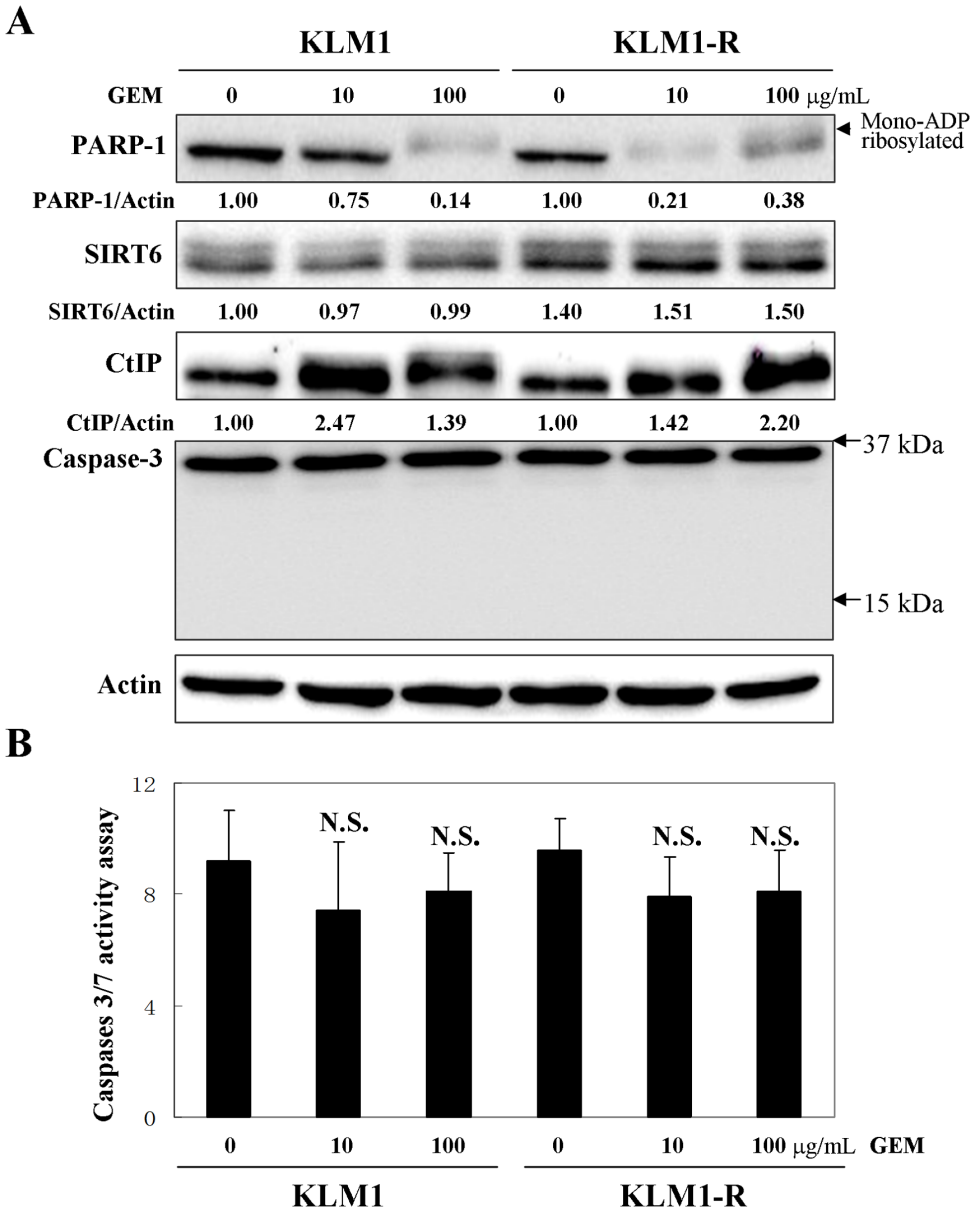
GEM down-regulates mono-ADP ribosylated PARP-1 in a caspase-independent manner. (A) KLM1 and KLM1-R cells were treated by GEM with the indicated concentrations for 24 h. Cell lysates were resolved in SDS-PAGE and probed with specific antibodies. The expression of PARP-1 was confirmed repeatedly by a distinct PARP-1 antibody described in Materials. An arrow head indicated the mono-ADP ribosylated form of PARP-1. Arrows indicated the position area of cleaved caspase-3. (B) The indicated cells were treated as in (A) and then stained using a caspases 3/7 assay kit (A). Caspase 3/7 activity was tested and measured by confocal microscopy and Image J. N.S., non significant. Error bars, SD.

We further tested the effect of GEM on PARP-1 expression in PC cells using a western blot analysis. KLM1 and KLM1-R showed a remarkable reduction of PARP-1 when cells were exposed to 10 or 100 μg/mL of GEM for 24 h (Fig. 2 A). Interestingly, it was observed that the reduced bands of PARP-1 in the GEM-induced panels showed a slight increase in molecular weight than in the untreated panels. Thus this suggested that GEM specifically induced the down-regulation of PARP-1 which was mono-ADP ribosylated (Fig. 2 A). Zhiyong Mao *et al.* had defined the upper band of PARP-1 as the mono-ADP ribosylated form at lysine residue 521 which was induced by sirtuin 6 (SIRT6) [26]. SIRT6 is a mammalian homolog of the yeast Sir2 deacetylase and involved in cytokine production and migration of PC [27]. Moreover, KLM1-R showed a higher sensitivity to GEM-induced down-regulation of PARP-1 than KLM1 cells. Ten μg/mL of GEM was enough to significantly reduce much of the PARP-1 expression in KLM1-R compared to KLM1 cells (Fig. 2 A). To find the explanation, we then compared the expression of SIRT6 between KLM1 and KLM1-R cells. Expectedly SIRT6 showed stronger expression (approximately 1.5-fold) in KLM1-R than KLM1 cells (Fig. 2 A). This indicated that the efficiency of GEM on the down-regulation of mono-ADP ribosylated PARP-1 may depend on the expression of SIRT6. We also observed that GEM induced the overexpression of CtBP-interacting protein (CtIP) in both KLM1 and KLM1-R cells (Fig 2 A). Because the caspase family protease cleaves the death substrate PARP-1 to a specific 85 kDa form observed during apoptosis [28], we next investigated whether caspase-3/7 were related to the PARP-1 down-regulation herein. We revealed that the level of caspase-3/7 activity as well as apoptosis showed no differences between KLM1 and KLM1-R cells even when cells underwent GEM treatment for 24 h as shown by western blotting (Fig. 2 A) and caspase-3/7 activity assay (Fig. 2 B). These data suggested that GEM specifically down-regulated mono-ADP ribosylated PARP-1 in a caspase-independent manner.

### GEM suppresses the expression of mono-ADP ribosylated PARP-1 through the autophagy degradation pathway

**Figure 3:**
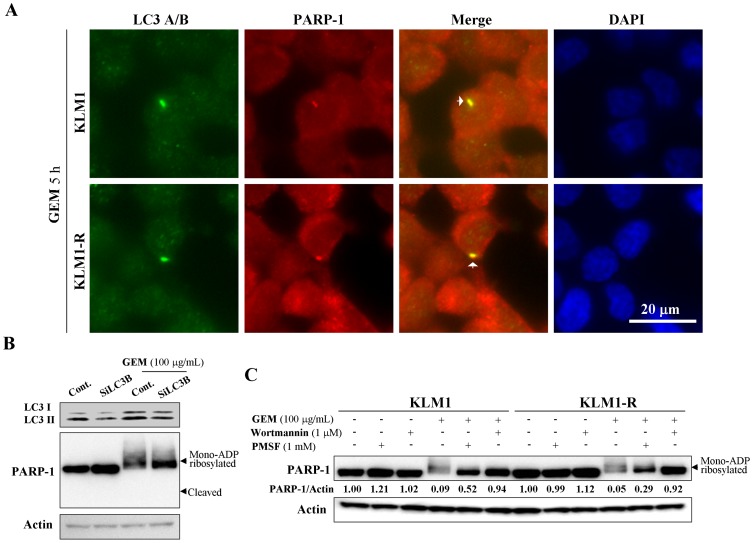
GEM suppresses mono-ADP ribosylated PARP-1 expression via autophagy. (A) KLM1 and KLM1-R cells were treated with 100 μg/mL of GEM for 5 h. Treated cells were stained with specific antibodies against LC3A/B and PARP-1 and then detected by confocal microscopy. LC3A/B: green, PARP-1: red and DAPI: blue. Scale bar, 20 μm. Arrows indicate the yellow staining of co-localizations between autophagosome and PARP-1. (B) KLM1 cells were exposed to GEM for 24 h after LC3B knockdown. (C) KLM1 and KLM1-R cells were exposed to GEM for 24 h in the present or absent of either PMSF or wortmannin at the indicated concentrations. Cell lysates were resolved by SDS-PAGE and probed with specific antibodies against to PARP-1. The arrow head indicates the mono-ADP ribosylated form of PARP-1. The expression of PARP-1 was confirmed repeatedly by a distinct PARP-1 antibody described in Materials.

As GEM induced autophagy and the mono-ADP ribosylated PARP-1 down-regulation in a caspase-independent manner, we investigated if mono-ADP ribosylated PARP-1 could be directly degraded by autophagy. KLM1 and KLM1-R cells were stained with both anti-LC and anti-PARP-1 antibody after cells were exposed to GEM for 5 h and then examined by immunofluorescent microscopy. GEM-induced autophagosome formation was found to co-localizate with PARP-1 in both KLM1 and KLM1-R cells (Fig. 3 A), indicating the relationship between autophagy and PARP-1. To test GEM-induced down-regulation of mono-ADP ribosylated PARP-1 through autophagy, LC3B siRNA, wortmannin (a PI3K inhibitor) and PMSF (a vacuolar protease inhibitor) were used to inhibit autophagy of cells in response to GEM. GEM induced PARP-1 mono-ADP ribosylation and down-regulation in KLM1, and the down-regulation of PARP-1 was reversed by LC3B knockdown (Figure 3 B). This reduction of PARP-1 expression by GEM in both KLM1 and KLM1-R cells were also rescued by treatment with either wortmannin or PMSF (Fig. 3 C). Together, these data demonstrated that GEM-induced down-regulation of mono-ADP ribosylated PARP-1 was mediated by the autophagy degradation pathway.

### Serum starvation induces activation and different localization of extracellular signal-regulated kinase (ERK) in KLM1 and KLM1-R cells

**Figure 4:**
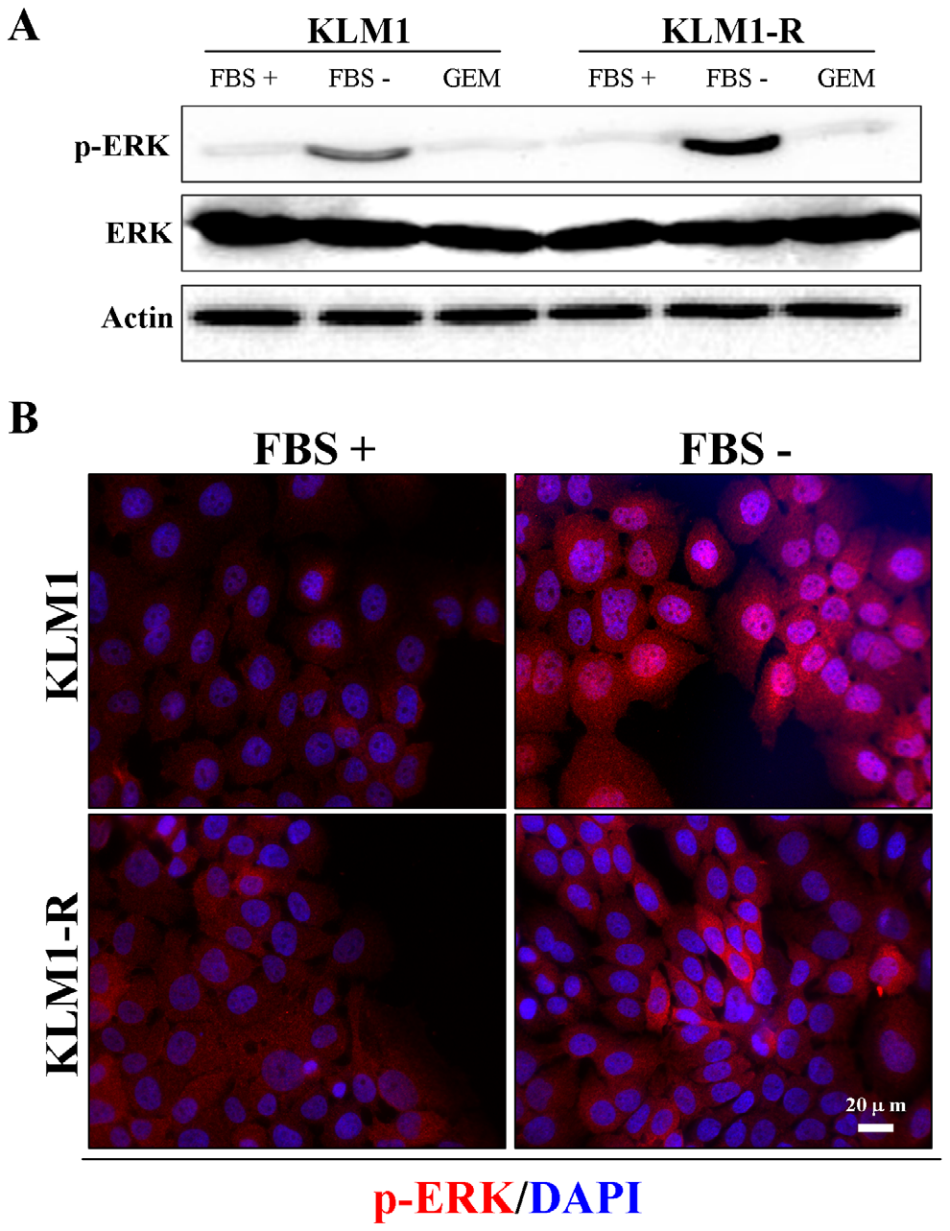
Serum starvation induces activation and different localization of extracellular ERK between KLM1 and KLM1-R cells. (A) KLM1 and KLM1-R cells were cultured in medium with or without FBS or exposed to 10 μg/mL of GEM for 24 h. Cell lysates were resolved by SDS-PAGE and probed with specific antibodies against p-ERK and ERK. (B) and (C) The indicated cells were stained with specific antibodies against p-ERK, Hsp27 and LC3A/B after cells were cultured in medium with or without FBS for 24 h. DAPI: blue and p-ERK: red in (B) and LC3A/B: green and Hsp27: red in (C). Scale bar, 20 μm.

To determine the effect of serum starvation on ERK activity and autophagy, cells were cultured in fresh medium with or without FBS for 24 hours and examined by western blot and immunofluorescent microscopy. We demonstrated that ERK was activated by serum starvation in KLM1 and KLM1-R cells and that GEM has no effect on ERK activity (Fig. 4 A). Next we examined the intracellular localization of phospho-ERK by immunofluorescence in KLM1 and KLM1-R cells. Interestingly, a remarkable difference in intracellular localization of p-ERK was shown between them. Under serum starvation, p-ERK was partially translocated into the nucleus in KLM1 cells; on the contrary, activated ERK was present solely in the cytoplasm in KLM1-R cells (Fig. 4 B). These results suggested that serum starvation induced activation of ERK in both of KLM1 and KLM1-R, but resulted in a difference in intracellular localization between them.

### Serum starvation suppresses GEM-induced PARP-1 degradation by inhibiting autophagy via the ERK signaling pathway

**Figure 5:**
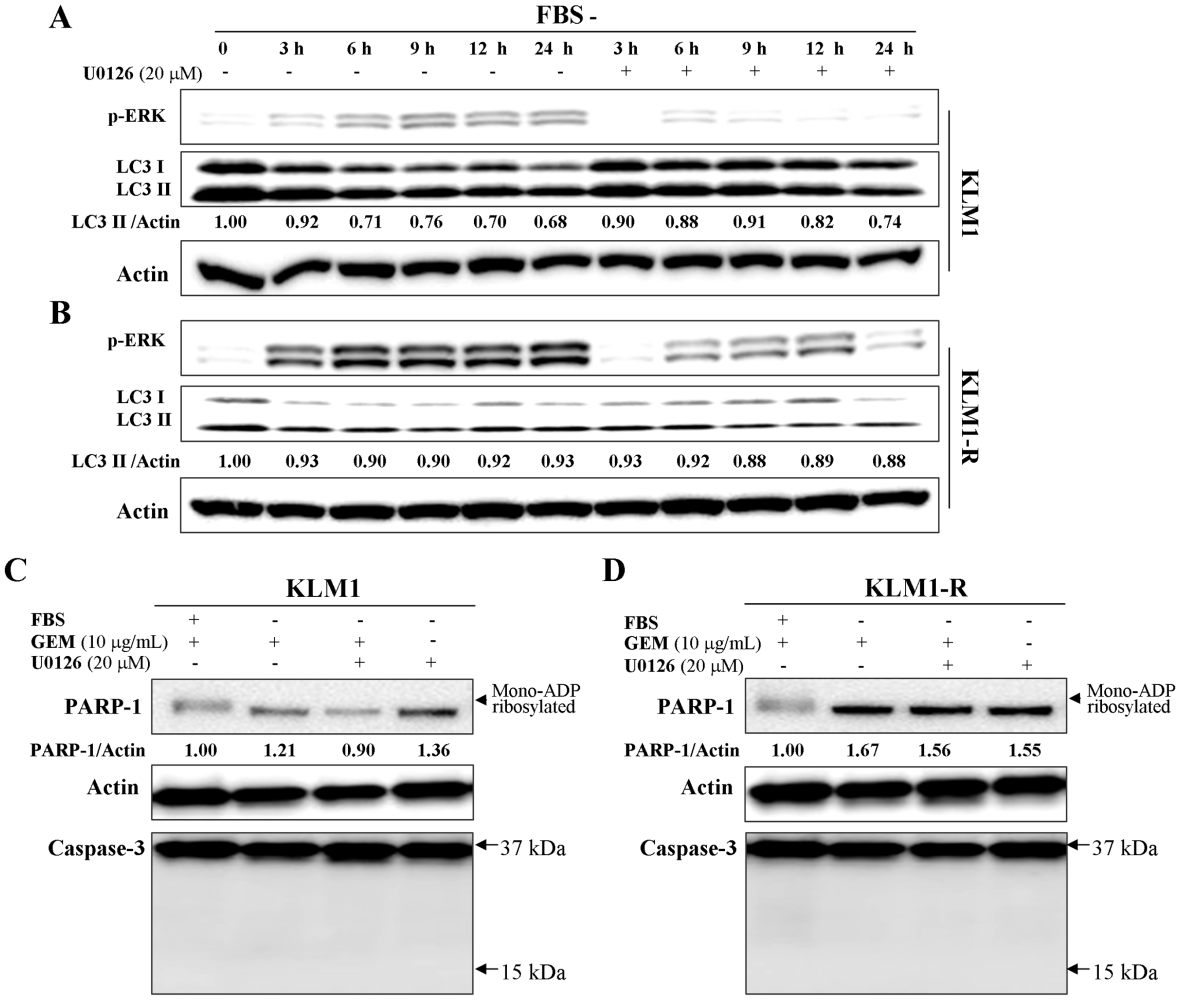
Serum starvation suppresses GEM-induced PARP-1 degradation through inhibition of autophagy via the ERK signaling pathway. (A) KLM1 and KLM1-R cells were exposed to 10 μg/mL of GEM in the presence or absence of 20 μM of U0126 for the indicated time courses. Cell lysates were resolved by SDS-PAGE and probed with specific antibodies against p-ERK and LC3A/B. (B) and (C) KLM1 and KLM1-R cells were cultured in the medium with or without FBS and meanwhile exposed to either or both GEM and U0126 at the indicated concentration. Cell lysates were resolved by SDS-PAGE and probed with specific antibodies. The arrow head indicates the mono-ADP ribosylated form of PARP-1. Arrows indicate the position area of cleaved caspase-3. The expression of PARP-1 was confirmed repeatedly by a distinct PARP-1 antibody described in Materials.

We next examined the autophagic activity after serum deprivation in KLM1 and KLM1-R cells in combination with an extracellular signal–regulated (ERK) kinase (MEK) inhibitor, U0126, to assess the effects of the MEK/ERK pathway on autophagy. We demonstrated that the expression of LC3 was reduced by serum starvation over a time course of 24 h in KLM1 and KLM1-R cells (Fig. 5 A and B) and rescued by U0126 in KLM1 (Fig. 5 A) but much less in KLM1-R (Fig. 5 B). The activity of ERK still showed an increasing trend in KLM1-R when treated with U0126 (Fig. 5 B), indicating a tolerance to U0126 in KLM1-R compared to KLM1 cells. This data indicated that serum starvation-induced autophagy inhibition was mediated by the MEK/ERK signaling pathway. Under serum starvation, U0126 had no effect on the expression of B-cell leukemia/lymphoma 2 (Bcl2) in KLM1 and KLM1-R cells and the reduction of p21 was delayed by treatment with U0126 in KLM1 but not in KLM1-R cells (Fig. S2 A and B), suggesting that the MEK inhibitor had different efficacy on the cell cycle progression between KLM1 and KLM1-R cells. Because the MEK inhibitor showed different efficacy on the modulation of autophagy between KLM1 and KLM1-R cells under serum starvation, we investigated its efficacy on the PARP-1 degradation in response to GEM. Indeed, GEM-induced PARP-1 degradation was inhibited by serum starvation in both KLM1 and KLM1-R cells in a caspase-independent manner and was reversed only in KLM1 cells by U0126 (Fig. 5 C and D). Moreover, the treatment by U0126 alone had no influence on PARP-1 expression. This data suggested that serum starvation suppresses autophagy and GEM-induced PARP-1 degradation through activation of the ERK signaling pathway, with KLM1 and KLM1-R cells showing different sensitivities to the MEK inhibitor U0126.

### Hypoxia suppresses autophagy and GEM-induced PARP-1 degradation

**Figure 6:**
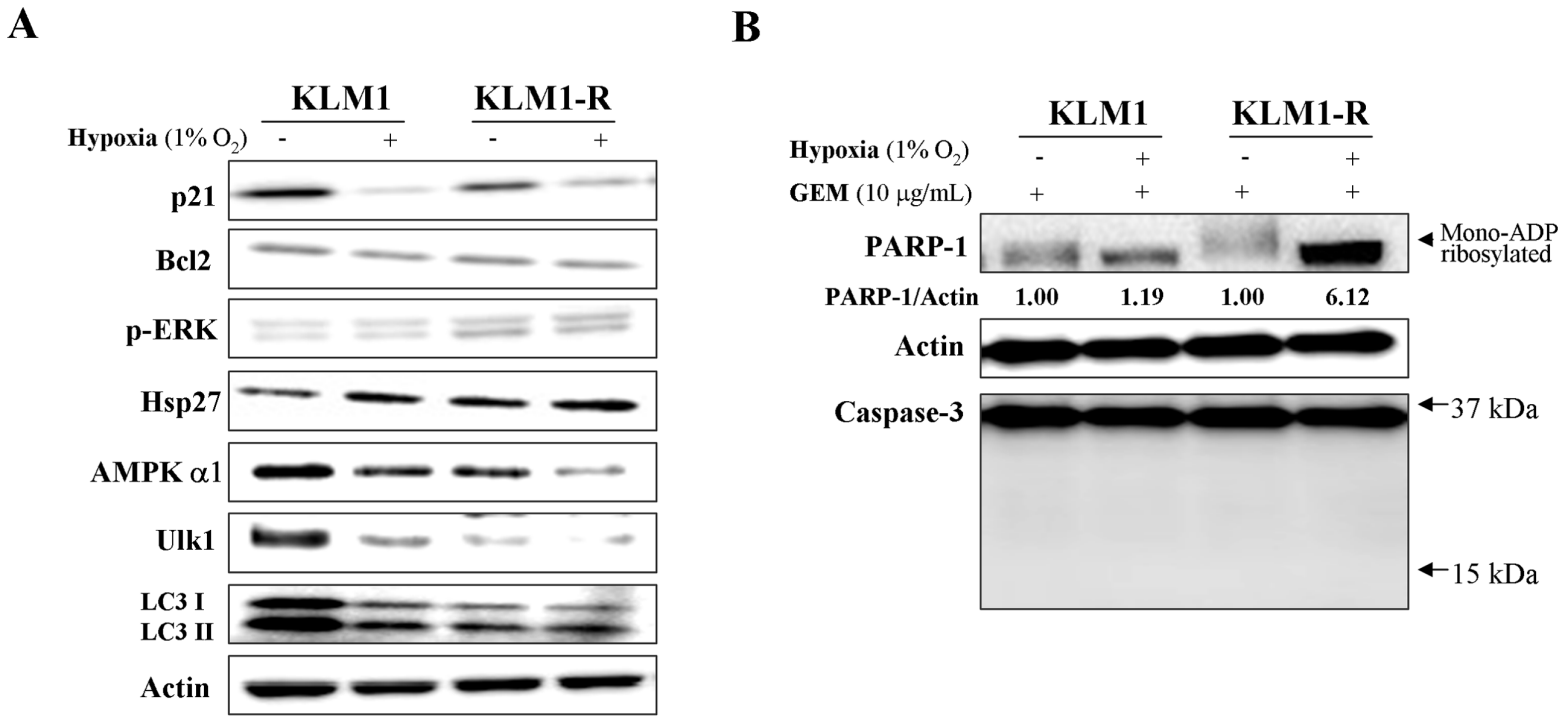
Hypoxia suppresses autophagy and GEM-induced PARP-1 degradation. (A) KLM1 and KLM1-R cells were cultured in normal conditions or 1% O_2_ hypoxia for 24 hours, and then cell lysates were resolved by SDS-PAGE and probed with specific antibodies. (B) The indicated cells were cultured in normal conditions or 1% O_2_ hypoxia together with 10 μg/mL of GEM for 24 hours. Cell lysates were resolved by SDS-PAGE and probed with specific antibodies. An arrow head indicates the mono-ADP ribosylated form of PARP-1. Arrows indicate the position area of cleaved caspase-3. The expression of PARP-1 was confirmed repeatedly by a distinct PARP-1 antibody described in Materials.

Hypoxia leads to cell cycle arrest via decreased p21 synthesis [29]. We confirmed that the expression of p21 was down-regulated and Hsp27 was up-regulated by 1% O_2_ hypoxia for 24 hours in KLM1 and KLM1-R cells; however, hypoxia had no influence on the expression of phospho-ERK and Bcl2 (Fig. 6 A). Under hypoxic condition, both AMPKα1 and Ulk1 expression were down-regulated in KLM1 and KLM1-R cells and the expression of LC3 in KLM1 was down to the same level as in untreated KLM1-R cells (Fig. 6 A), indicating that hypoxia induced phenotypic change in KLM1 leading to a KLM1-R-like condition and inhibition of autophagy. Thus we tested if hypoxia inhibited GEM-induced PARP-1 degradation. Western blot analysis demonstrated that GEM-induced PARP-1 degradation was remarkably abolished by hypoxia in a caspase-independent manner in both KLM1 and KLM1-R cells (Fig. 6 B). These results indicated that hypoxia suppresses GEM-induced PARP-1 degradation by reducing autophagic activity.

## Discussion

Autophagy can be elevated by GEM in the treatment of PC cells [13]. PC cells showed to be more sensitive to the cytotoxic effect of GEM when this was combined with cannabinoids via reactive oxygen species (ROS)-mediated autophagic cell death [30]. In this study, we demonstrated that autophagic activity was reduced in GEM-resistant KLM1-R compared to -sensitive KLM1 cells. Therefore, reactivation of autophagy might be a useful strategy for resensitising PC to GEM. However little is known about the role of autophagy in DNA damage response induced by GEM. There is important evidence that autophagy is associated with the processing of double-strand breaks (DSBs) and cell death in response to DNA damage in yeast through degradation of acetylated recombination protein Sae2 (human CtIP) [31]. Inhibition/ablation of histone deacetylases (HDACs) induces autophagy and acetylation of a number of DNA damage response (DDR) proteins, including Sea2 and Exo1 [31], [32]. Here we show that GEM induces a DNA damage response (observed through CtIP overexpression by western blot) and mono-ADP ribosylated PARP-1 degradation leading to increased autophagy. Thus autophagy involved in DNA damage repair may be through the control of PARP-1 degradation rather than CtIP (yeast Sae2) in human PC. However, whether the mono-ADP ribosylation of PARP-1 is necessary for autophagy degradation and how this specific degradation is implemented in response to GEM should be clarified in further study.

Ablation of PARP-1 does not interfere with DSBs repair, but delays reactivation of stalled replication forks [33]. Therefore, GEM-induced degradation of PARP-1 may contribute to GEM-stalled replication forks. DSB response factors ATM, Mre11, and Rad50 are required for cell survival after replication fork stalling in response to GEM-induced DNA damage [34]. Restart of stalled replication forks and repair of collapsed replication forks require RAD51 activity and RAD51-mediated homologous recombination (HR) pathway, respectively [35]. Moreover, we demonstrate that CtIP is overexpressed in response to GEM in KLM1 and KLM1-R cells (Fig. 2 A). Taken together, these results suggest that DSBs repair is required for survival of cells as well as PC cells after GEM-induced DNA damage. CtIP was shown to be up-regulated in both KLM1 and KLM1-R induced by GEM, but its stability seems stronger in KLM1-R, particularly over a concentration rage of 10–100 μg/mL GEM, which expresses a higher level of SIRT6 compared to KLM1 cells (Fig. 2 A). SIRT6 promotes DNA stability and activation and stabilization of CtIP by deacetylation [31], [35], indicating a possible mechanism for the lower sensitivity of KLM1-R to GEM compared to KLM1 cells. Moreover, recent studies suggest that PARP inhibitors are particularly lethal to cells deficient in homologous recombination (HR) proteins through deregulation of error-prone non-homologous end joining [36]. Likewise, therefore, combination of a kind of HR inhibitor with GEM (as a PARP-1 suppressor) may be a potential therapeutic strategy for PC. However, there is a major limitation because serum starvation and hypoxia (mimicking tumor microenvironments *in-vivo*) inhibit GEM-induced PARP-1 degradation by reducing autophagic activity (Fig. 5 and 6). Thus, the preferred candidate for combination therapy with GEM should not only inhibit the components of DSBs but also promote autophagy, for example a histone deacetylases inhibitor, namely, valproic acid [37]. Further studies are needed to test whether this inhibitor could enhance PC cell death in response to GEM.

The MEK inhibitor U0126 shows different effects on the level of autophagy and PARP-1 degradation in response to GEM between KLM1 and KLM1-R cells, indicating that possibly limitations exist on the therapeutic strategy for targeting the EGFR/Ras/ERK pathway in PC. It is presumed that the observed differences depend on one of three possibilities: 1) the differences in intracellular localization of activated ERK between KLM1 and KLM1-R cells (Fig. 4 B); 2) an unknown feedback signaling pathway for ERK reactivation in response to the MEK inhibitor (Fig. 5 B) [37], [38]; 3) serum-induced down-regulation of ULK1 in KLM1-R cells leading to autophagy not controlled by ERK (Fig. S1 B).

In this study, we reveal new highlights that GEM functions as a suppressor of PARP-1 by promoting the autophagy degradation pathway and put forward the related suggestions about the desired characteristics of possible candidates for combination therapy with GEM for PC.

## Supporting Information

Figure S1 **(A) KLM1 and KLM1-R cells were lysed and resolved in SDS-PAGE and probed with specific antibodies.** Actin was used to normalize the loading levels of protein. (B) KLM1 and KLM1-R cells were cultured in medium with or without FBS or exposed to 10 μg/mL of GEM for 24 h. Cell lysates were resolved in SDS-PAGE and probed with specific antibodies.
(TIF)

Figure S2 **KLM1 (A) and KLM1-R (B) cells were exposed to 10 μg/mL of GEM in present or absent of 20 μM of U0126 for the indicated time courses.** Cell lysates were resolved in SDS-PAGE and probed with specific antibodies against to p21 and Bcl2. The relative intensities of western blot were measured and shown in this figure.
(TIF)
