# Supplementary figures and images for: Gemcitabine Induces Poly (ADP-Ribose) Polymerase-1 (PARP-1) Degradation through Autophagy in Pancreatic Cancer
Source: PLoS One. 2014 Oct 1;9(10):e109076. doi: 10.1371/journal.pone.0109076 (PMC4182782; doi:10.1371/journal.pone.0109076)

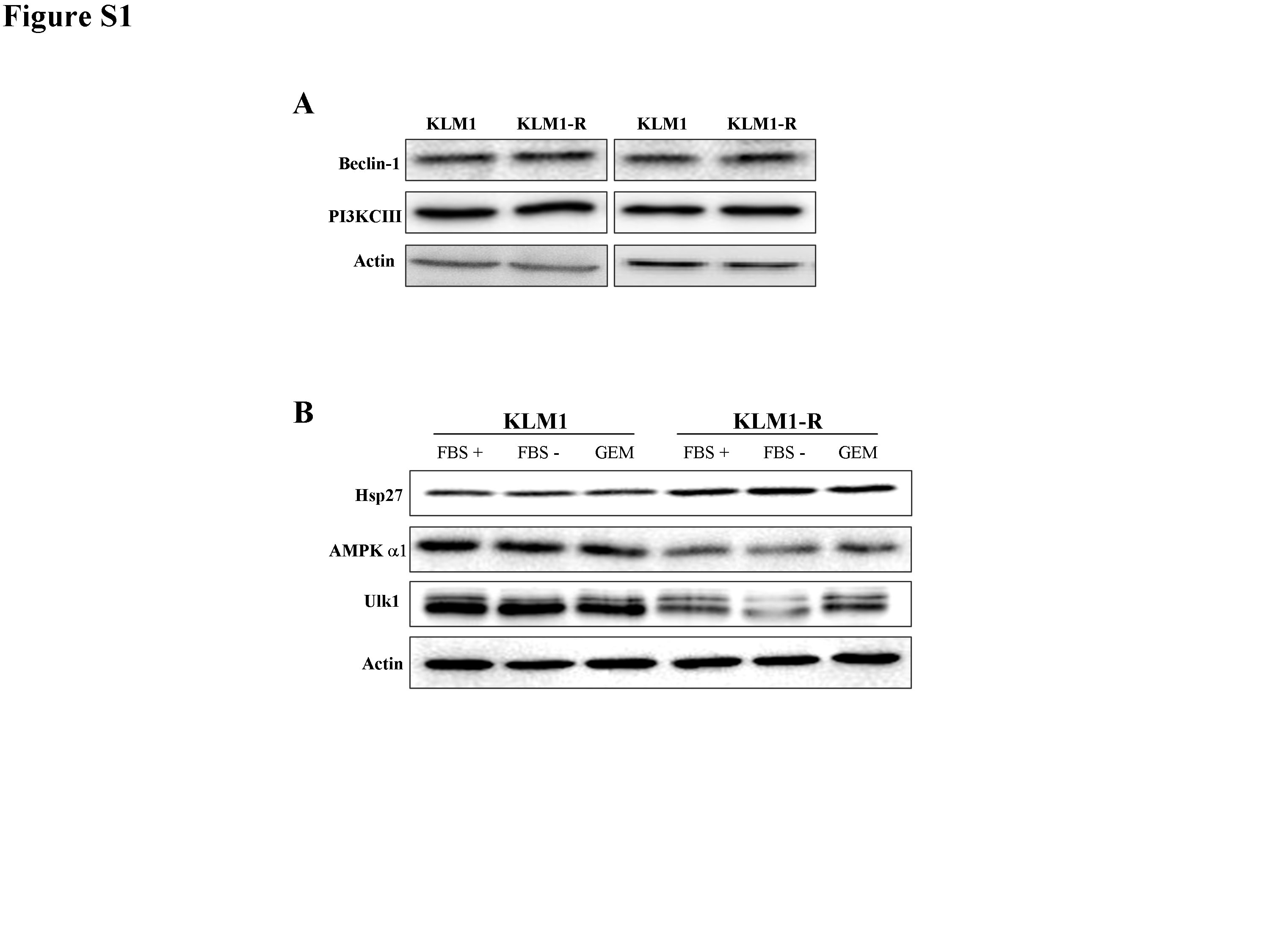

Supplement: Figure S1 — (A) KLM1 and KLM1-R cells were lysed and resolved in SDS-PAGE and probed with specific antibodies. Actin was used to normalize the loading levels of protein. (B) KLM1 and KLM1-R cells were cultured in medium with or without FBS or exposed to 10 μg/mL of GEM for 24 h. Cell lysates were resolved in SDS-PAGE and probed with specific antibodies. (TIF) [file pone.0109076.s001.tif]

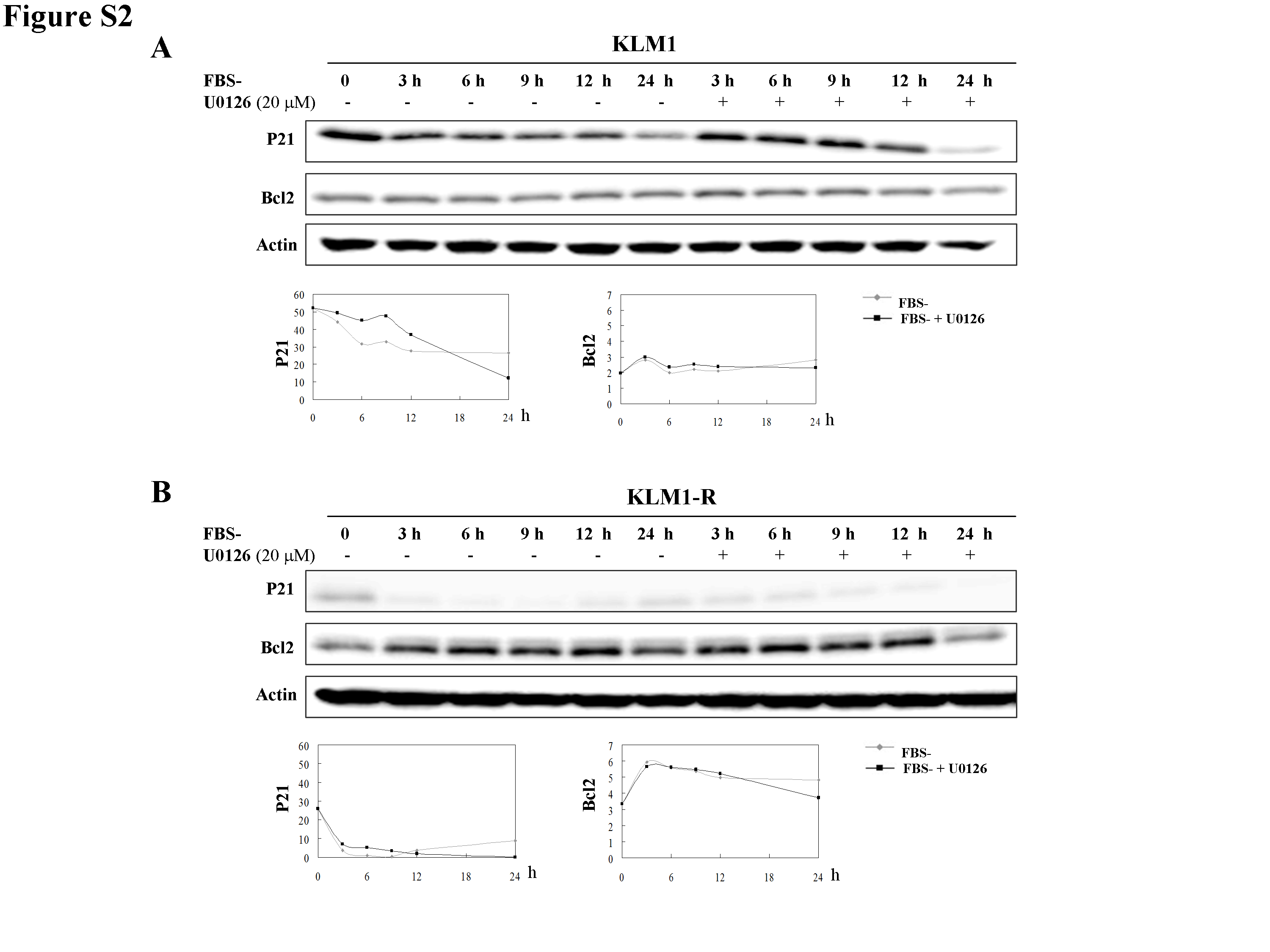

Supplement: Figure S2 — KLM1 (A) and KLM1-R (B) cells were exposed to 10 μg/mL of GEM in present or absent of 20 μM of U0126 for the indicated time courses. Cell lysates were resolved in SDS-PAGE and probed with specific antibodies against to p21 and Bcl2. The relative intensities of western blot were measured and shown in this figure. (TIF) [file pone.0109076.s002.tif]
